# Therapy of moderate-to-severe Graves’ orbitopathy with intravenous methylprednisolone pulses is not associated with loss of bone mineral density

**DOI:** 10.1007/s12020-018-1823-x

**Published:** 2018-12-01

**Authors:** Joanna Rymuza, Michał Popow, Zuzanna Żurecka, Jerzy Przedlacki, Tomasz Bednarczuk, Piotr Miśkiewicz

**Affiliations:** 1 Department of Internal Medicine and Endocrinology, Medical University of Warsaw, Banacha 1a, 02-097, Warsaw, Poland; 2 Department of Nephrology, Dialysis and Internal Medicine, Medical University of Warsaw, Banacha 1a, 02-097, Warsaw, Poland

**Keywords:** Bone mineral density, Gucocorticoid-induced Osteoporosis, Methylprednisolone, Graves’ ophthalmopathy, Graves’ orbitopathy, Trabecular bone score

## Abstract

**Purpose:**

To evaluate the influence of intravenous methylprednisolone (IVMP) pulse administration on bone mineral density (BMD) of the lumbar spine and the femoral neck in patients with moderate-to-severe Graves’ orbitopathy (GO).

**Methods:**

Thirty-five patients with GO in euthyreosis were treated with 12 IVMP pulses (6 × 0.5 g, 6 × 0.25 g on a weekly schedule). Supplementation with 1.0 g of calcium and 800 IU of vitamin D was initiated in all patients before beginning therapy. BMD of the lumbar spine (L1–L4) and the femoral neck were assessed at baseline and after the last IVMP pulse using dual-energy X-ray absorptiometry. To determine differences in BMD between values at baseline and after treatment, we used the least significant change (LSC) methodology. LSC values were calculated to be 3 and 5% for the lumbar spine and the femoral neck, respectively. Change in BMD equal to or exceeding the LSC was assessed as either increase or decrease of BMD. We then compared pre-treatment and post-treatment mean BMD values at the lumbar spine and the femoral neck.

**Results:**

We did not observe a decrease of BMD at any site equal to or exceeding the LSC. We found an increase of BMD in at least one measurement site equal to or exceeding the LSC value in 43% of patients, mostly in the lumbar spine (31%). Mean femoral neck BMD did not change while mean lumbar BMD increased.

**Conclusions:**

IVMP given in weekly intravenous pulses does not lead to loss of BMD of the lumbar spine and the femoral neck.

## Introduction

Therapy with high dose intravenous glucocorticoids (GCs) is an effective immunosuppressive treatment used in various inflammatory and autoimmune diseases [1]. Intravenous methylprednisolone (IVMP) pulse therapy is still considered to be the standard treatment in patients with active, moderate-to-severe and very severe Graves’ orbitopathy (GO) [2]. This therapy is recommended by the European Group on Graves’ Orbitopathy (EUGOGO) due to higher efficacy and fewer adverse effects compared to oral GCs [1–4]. There are studies, however, which report side effects associated with this therapy (e.g., pulmonary embolism, myocardial infarction, severe cerebrovascular events, acute liver damage and sudden death, as well as changes in coagulation status and blood pressure) [1, 2, 4–8]. One of the most serious side effects of long-term treatment with GCs is osteoporosis [9–11]. However, knowledge about the deleterious effects of GCs on bone comes mostly from studies involving patients taking oral GCs [11, 12]. GCs impact bone cells directly, increasing their rate of apoptosis and thereby shortening the lifespan of osteoblasts and osteocytes [9, 13, 14]. At the same time the opposite effect is taking place in osteoclasts, resulting in their prolonged lifespan [13, 14]. Secondly, an excess of GCs stimulates the mineralocorticoid receptor within parathyroid chief cells [15]. The mineralocorticoid receptor stimulation impacts parathyroid hormone (PTH) secretion by the chief cells leading to an overall increase in PTH levels [15]. The bone building, anabolic effects require brief exposures to higher than average PTH concentrations [16]. The catabolic effects, on the other hand, result from continuous excessive secretion of PTH leading to bone destruction [16, 17]. GCs also provoke decreased calcium absorption in the gut [18]. This decreased absorption of calcium results in hypocalcemia, which feeds back and further stimulates PTH secretion [15]. Summarizing, the effect of GCs on bone differs depending on the duration and dosage of therapy, as well as its route of administration [19].

Data in literature regarding the influence of intravenous GCs on BMD are limited and inconclusive (Table 1). Some studies indicate a lack of adverse effects of intravenous GCs on BMD [20–22] or even an increase in BMD [22]. Others, however, report loss of BMD [23, 24]. To our knowledge the impact of IVMP on BMD in patients with GO has not been previously analysed. The aim of our study was to evaluate the influence of IVMP pulse therapy on BMD in euthyroid patients with moderate-to-severe GO. We hypothesized that BMD of the lumbar spine and the femoral neck would not be changed after therapy with high dose intravenous GCs given according to a weekly schedule.

**Table 1:** Summary of studies that investigated changes in BMD in patients treated with IVMP

Study	Size of the study group (*n*)	Diagnosis	IVMP regimen and concomitant treatment	BMD evaluation	Results
Schwid et al. [22]	30	MS	1.0 g of IVMP for 3 alternate days followed by oral GCs in tapering doses for 2 weeks. Cumulative dose of IVMP: 3.0 g No calcium and vitamin D supplementation	At baseline and after 2, 4, and 6 months	Increase of mean BMD values at lumbar spine No change of mean BMD values at femoral neck
Frediani et al. [20]	31	RA	1.0 g of IVMP for 3 alternate days repeated at monthly check if needed, with a cumulative dose of IVMP equal to 18.9 g (±4.2) for each patient in one year No calcium and vitamin D supplementation	At baseline and every 3 months for one year	No change of mean BMD values at lumbar spine, femoral neck and total-body
Dovio et al. [21]	13	MS	15 mg/kg of IVMP daily for 10 days No calcium and vitamin D supplementation	At baseline and after 6 months	No change of mean BMD values at lumbar spine, femoral neck ant total-body
Haugeberg et al. [24]	38	SLE, SS, GPA, RA, Miscellaneous	3.0 g (±1.6 g) of IVMP given as 5.7 (±2) pulses over a median period of 5.7 months Concomitant treatment with oral GCs, bisphosphates, estrogen in part of the group of patients Supplementation with calcium and vitamin D in part of the group of patients	At baseline and after 6 months	Decrease of mean BMD values at the lumbar spine, femoral neck, and total hip
Natsui et al. [23]	19	SLE, DM, PM, MCTD, GO, NS	4.5–12 g of IVMP in different regimens followed by oral GCs for 2 months No calcium and vitamin D supplementation	At baseline and after 2 months	Decrease of mean BMD values at lumbar spine, femoral neck and total-body
Current study	35	GO	Cumulative dose of 4.5 g of IVMP, divided into 12 weekly infusions (6 weekly infusions of 0.5 g, followed by 6 weekly infusions of 0.25 g) Supplementation with 1.0 g of calcium and 800 IU of vitamin D	At baseline and after 3 months	Increase of mean BMD values at lumbar spine No change of mean BMD values at femoral neck Increase in BMD in at least one measurement site equal to or exceeding the LSC value in 15 out 35 patients (43%)

*RA* rheumatoid arthritis, *MS* multiple sclerosis, *SLE* systemic lupus erythematosus, *DM* dermatomyositis, *PM* polymyositis, *MCTD* mixed connective tissue disease, *GO* Graves’ orbitopathy, *NS* nephrotic syndrome, *SS* systemic sclerosis, *GPA* granulomatosis with polyangiitis, *IVMP* intravenous methylprednisolone, *GCs* glucocorticosteroids, *BMD* bone mineral density, *LSC* least significant change

## Materials and methods

### Patients

The study was conducted at one academic referral center at the Medical University of Warsaw (WUM). Patients with active, moderate-to-severe GO were consecutively recruited from the Department of Endocrinology, WUM from 2012 to 2017. The diagnosis of GO was based on EUGOGO recommendations [2]. The study included 35 patients: 31 patients with Graves’ disease and 4 patients with Hashimoto thyroiditis. Twenty-one patients were treated with antithyroid drugs (alone or according to a “block and replace” schedule) and 13 patients received levothyroxine: 9 patients with Graves’ disease who were at least 6 months after the last radical treatment (6 patients after radioiodine therapy and 3 patients after thyroidectomy) and 4 patients with Hashimoto thyroiditis. One patient had euthyroid Graves’ disease. All patients remained clinically euthyroid, with free tri-iodothyronine (fT3) and free thyroxine (fT4) levels within the reference range at least one month preceding, as well as during the study. Exclusion criteria were: (i) treatment with GCs within the last six months, (ii) any other treatment known to significantly alter bone metabolism (e.g., bisphosphonates or other drugs with anti-fracture effects, heparin, vitamin-K antagonists), (iii) elevated, basal PTH levels, (iv) clinical diagnosis of osteoporosis based on BMD measurements and the presence of fractures, as defined by the World Health Organization [25]. The study was approved by the Local Bioethics Committee and was conducted in accordance with the Declaration of Helsinki. Written informed consent was obtained from all individual participants included in the study. The clinical characteristics of the analyzed group are shown in Table 2.

**Table 2:** Baseline characteristics of patients (*n* = 35)

	Number of patients (%) or mean ± SD (range)
*Thyroid disease*	
Graves’ disease treated for hyperthyroidism	21 (60%)
Graves’ disease after radical treatment on levothyroxine	9 (26%)
Euthyroid graves’	1 (3%)
Hashimoto thyroiditis on levothyroxine	4 (11%)
Age (years)	47 ± 12 (22–66)
*Sex*	
Women	29 (83%)
Men	6 (17%)
Height (m)	1.66 ± 0.08 (1.54–1.83)
Body mass index (kg/m^2^)	27 ± 4 (19–40)
Duration of thyroid disease (months)	48 ± 75 (4–384)
Duration of euthyreosis before IVMP (months)^a^	3 ± 2.4 (1–11)
Smokers	16 (46%)
TSH (normal range: 0.27–4.2 µIU/mL)	1.1 ± 1.2 (0.001–4.0)
fT4 (normal range 12.0–22.0 pmol/L)	16.3 ± 2.9 (12–21.4)
fT3 (normal range: 3.1–6.8 pmol/L)	4.9 ± 0.9 (3.6–6.6)
25(OH)D (ng/mL)	19.3 ± 8.8 (4.5–39.7)
DXA lumbar spine: BMD (g/cm^2^)	1.028 ± 0.11 (0.81–1.258)
DXA lumbar spine: *T*-score	−0.25 (−2.2 to +1.9)
DXA lumbar spine: *Z*-score	0.39 (−1.2 to +2)
DXA femoral neck: BMD (g/cm^2^)	0.846±0.12 (0.642–1.141)
DXA femoral neck: *T*-score	−0.21(−1.9 to +1.7)
DXA femoral neck: *Z*-score	0.52 (−0.8 to +2.4)

*TSH* thyroid-stimulating hormone, *fT4* free thyroxine, *fT3* free triiodothyronine, *25(OH)D* 25-hydroxyvitamin D, *IVMP* intravenous methylprednisolone, *DXA* dual-energy X-ray absorptiometry, *BMD* bone mineral density

^a^Duration of euthyreosis before IVMP is presented for 21 patients with Graves’ disease on thyreostatics

### Study design

All patients received IVMP pulses according to EUGOGO recommendations: cumulative dose of IVMP 4.5 g, treatment duration 12 weeks with once-weekly intravenous pulses, first 6 weeks 0.5 g IVMP, next 6 weeks 0.25 g of IVMP [2].

BMD of the lumbar spine (L1–L4) and the femoral neck was measured at baseline and after the last IVMP pulse using dual-energy X-ray absorptiometry (DXA). All DXA scans were performed by one technician using the same equipment (Hologic Discovery A Densitometer) and subsequently analyzed by the same physician. The DXA measurements were expressed as BMD (g/cm^2^). *Z*-scores and *T*-scores were subsequently calculated. According to the World Health Organization’s definitions, osteopenia was diagnosed in patients with a *T*-score between −1.0 and >−2.5 standard deviation (SD), and osteoporosis in those with a *T*-score of the lumbar spine and/or the femoral neck ≤−2.5 SD [25]. To determine whether differences in BMD between values at baseline and after treatment in each individual patient were significant, we used the least significant change (LSC) methodology. LSC values were determined for the DXA machine in the Medical University of Warsaw’s densitometry lab and were calculated to be 3 and 5% for the lumbar spine and the femoral neck respectively. Change in BMD equal to or exceeding the LSC was assessed as either increase or decrease of BMD. In addition, we compared pre-treatment and post-treatment mean BMD values at the lumbar spine and the femoral neck. Height assessment of the patients was performed before and after therapy with IVMP pulses.

Serum levels of calcium (Ca), phosphate (P), intact parathyroid hormone (iPTH), 25-hydroxyvitamin D [25(OH)D], thyroid-stimulating hormone (TSH), fT3 and fT4 were assessed before the 1st and 12th IVMP pulse. According to the guidelines for vitamin D supplementation and treatment of deficits approved in Central Europe [26], we defined concentrations below 20 ng/mL as vitamin D deficiency, concentrations of 20–30 ng/mL as suboptimal vitamin D status and concentrations higher than 30 ng/mL as optimal vitamin D status. At baseline vitamin D deficiency was observed in 18 patients (51%, mean 19.3 ng/mL). Supplementation with 1.0 g of calcium and 800 IU of vitamin D was routinely initiated in all patients on the first day of IVMP therapy and continued throughout the observation period.

Calcium and phosphate were analyzed calorimetrically. We measured iPTH, 25(OH)D, TSH, fT4, and fT3 using an electrochemiluminescence immunoassay on Cobas 8000 Analyzer (Roche Diagnostics, Mannheim, Germany). The normal ranges were as follows: Ca, 2.15–2.6 mmol/L; P, 0.81–1.45 mmol/L; iPTH, 15–65 pg/mL; TSH, 0.27–4.2 µIU/mL; fT3, 3.1–6.8 pmol/L; fT4, 12.0–22.0 pmol/L.

### Statistical analysis

All analyses were performed using SPSS statistical software version 22.0 (IBM SPPS Statistics, New York, US). Continuous variables are expressed as means ± SD, while categorical variables are expressed as numbers (*n*) and percentages (%). The Shapiro-Wilk test was used to confirm or reject the normal distribution of each continuous variable. Comparisons between continuous data were performed using paired *t*-test (for parameters with normal distribution) or Wilcoxon rank sum test (for parameters with distribution deviations). Chi-squared or Fisher exact test was used to analyze the differences between categorical data. Pearson correlation test was performed to investigate correlations. Statistical significance was established for results with *p* value <0.05.

## Results

### Effect of IVMP treatment on BMD

**Fig. 1:**
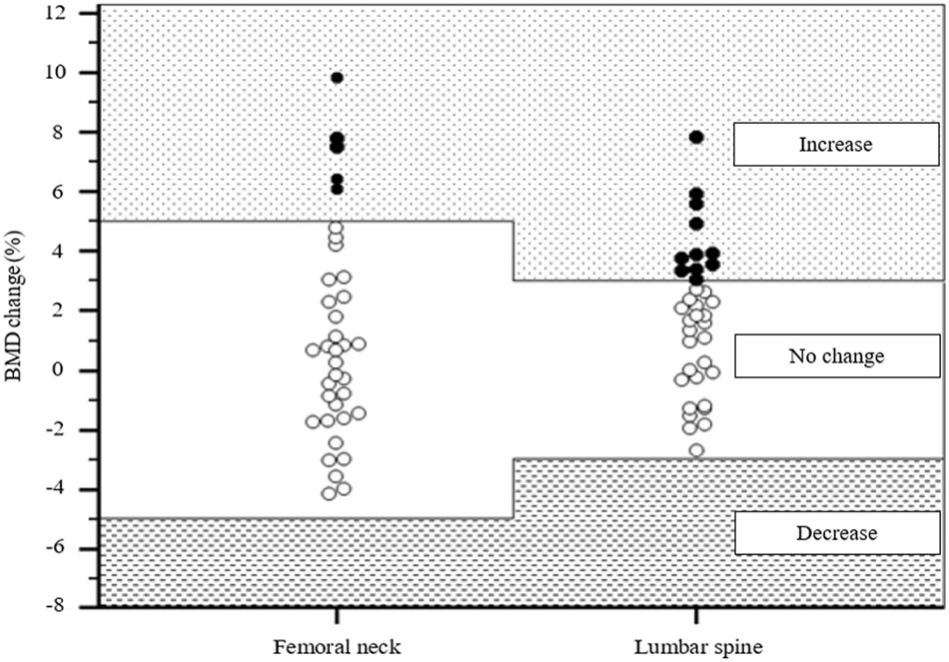
Percentage of BMD change in the femoral neck and the lumbar spine according to LSC criteria in 35 patients after therapy with IVMP pulses. Bullets represent individual percentage of BMD changes (black bullets represent an increase in BMD—equal to or exceeding LSC, calculated to be 5% change for femoral neck and 3% change for lumbar spine; white bullets represent no change in BMD). BMD–bone mineral density

According to the LSC criteria, no one in the group experienced a decrease of BMD at any site equal to or exceeding the LSC value following 12 weeks of IVMP. We observed an increase of BMD in at least one measurement site equal to or exceeding the LSC value in 15 out of 35 patients (43%): in 11 out of 35 patients (31%) with increased lumbar spine BMD and in 5 out of 35 patients (14%) with increased femoral neck BMD (Fig. 1). In one patient out of 35 (3%) an increase of BMD in both measurement sites was noted. The results are not different if only postmenopausal women are being considered. We observed an increase of BMD in at least one measurement site equal to or exceeding the LSC value in 5 out of 12 postmenopausal women (42%).

Mean lumbar BMD increased after the last IVMP pulse, becoming 1.7% greater than baseline (*p* value = 0.0003). At the femoral neck, however, there was no significant change in mean post-treatment BMD as compared to the baseline (Table 3). Analyzing a subgroup of postmenopausal women, mean femoral neck BMD did not change while mean lumbar BMD increased by 2.2% (*p* value = 0.02).

**Table 3:** Changes in BMD after 12 weeks of IVMP treatment

	Pre-treatment	Post-treatment	*p* value
Lumbar spine (L1–L4)	1.028 ± 0.11	1.045 ± −0.11	0.0003
Femoral neck	0.642 ± 0.12	0.671 ± 0.12	0.19

*BMD* bone mineral density, *IVMP* intravenous methylprednisolone

There were no significant differences between the groups with increased BMD (gain in BMD equal or exceeding the LSC) vs. no change in BMD after IVMP treatment as far as the selected characteristics were considered (Table 4).

**Table 4:** Comparison of selected characteristics of patients with and without increase of BMD (gain in BMD equal to or exceeding the LSC) in the lumbar spine and/or femoral neck after IVMP therapy

	Increase of BMD 15/35 (43%)	No change of BMD 20/35 (57%)	*p* value
Age (years), mean±SD	51 ± 14	46 ± 11	0.77
Women, *n* (%)	12 (80%)	17 (85%)	1.00
Women after menopause, *n* (%)	5 (33%)	7 (35%)	1.00
BMI (kg/m^2^), mean±SD	26 ± 4	26 ± 5	0.67
Duration of GO (months), mean ± SD	13 ± 38	21.5 ± 92	0.13
Duration of euthyreosis before IVMP (months), mean ± SD^a^	2.6 ± 1.4^b^	2.8 ± 2.9^c^	0.31
Smokers, *n* (%)	7 (47%)	8 (40%)	0.74
TSH (µIU/mL), mean ± SD	1.1 ± 1.1	1.4 ± 1.3	0.43
Baseline 25(OH)D (ng/mL), mean ± SD	20.1 ± 10	18.5 ± 7	0.27
Vitamin D deficiency, *n* (%)	9 (60%)	9 (45%)	0.50
Vitamin D suboptimal level, *n* (%)	4 (27%)	9 (45%)	0.31
Vitamin D optimal level, *n* (%)	2 (13%)	2 (10%)	1.00
Osteopenia (−1.0 to >−2.5) before IVMP, *n* (%)	5 (33%)	7 (35%)	1.00

Values are presented as mean or as otherwise indicated

*BMD* bone mineral density, *BMI* body mass index, *TSH* thyroid-stimulating hormone, GO Graves’ orbitopathy, *IVMP* intravenous methylprednisolone, *25(OH)D* 25-hydroxyvitamin D, Vitamin D deficiency 25(OH)D level below 20 ng/mL, Vitamin D suboptimal level 25(OH)D level 20–30 ng/mL, Vitamin D optimal level 25(OH)D level above 30 ng/mL

^a^Duration of euthyreosis before IVMP is presented for 21 patients with Graves’ disease using thyrostatic drugs

^b^Mean duration of euthyreosis before IVMP assessed in 12 out of 21 former hyperthyroid patients in whom the increase of BMD was found

^c^Mean duration of euthyreosis before IVMP assessed in 9 out of 21 former hyperthyroid patients in whom the increase of BMD was not found

There were no significant correlations between changes in lumbar or femoral BMD and either baseline 25(OH)D status or 25(OH)D change. No relationship was found between the duration of euthyreosis before IVMP therapy in former hyperthyroid patients on antithyroid drugs and change in BMD of the femoral neck or lumbar spine. Details are presented in Tables 6 and 7 in the Supplementary Material.

We did not observe any variations in patients’ height after treatment.

### Effect of IVMP on serum Ca, P, iPTH, and 25(OH)D

We did not observe changes in serum Ca, P, and iPTH levels between the first and last IVMP pulses (Table 5). There was a significant increase in 25(OH)D concentration at the end of the study up to a mean level of 21.8 ng/mL (*p* value = 0.04). 5 out of 35 patients (14%) achieved optimal vitamin D status.

**Table 5:** Changes in serum calcium, phosphate, iPTH and 25(OH)D levels during IVMP treatment

	Before 1st IVMP pulse	Before 12th IVMP pulse	*p* value
Ca (mmol/L), mean±SD	2.35 ± 0.1	2.34 ± 0.1	0.31
P (mmol/L), mean±SD	1.16 ± 0.2	1.09 ± 0.1	0.15
iPTH (pg/mL), mean±SD	42.4 ± 19.4	44.1 ± 18.0	0.14
25(OH)D (ng/mL), mean±SD	19.3 ± 8.8	21.8 ± 7.6	0.04

*Ca* calcium, *P* phosphate, *iPTH* intact parathyroid hormone, *25(OH)D* 25-hydroxyvitamin D, *IVMP* intravenous methylprednisolone

## Discussion

Our prospective study evaluated influence of IVMP pulse therapy on BMD in patients with GO. We found that GCs given intravenously in weekly infusions do not lead to the loss of BMD of the lumbar spine and the femoral neck. On the contrary, we observed an increase of BMD (according to the LSC criteria) in at least one measurement site in 43% of patients (mostly in the lumbar spine), as well as an overall increase in mean BMD in the lumbar spine. These results are consistent with previous study concerning multiple sclerosis patients in which IVMP treatment (1.0 g for three alternate days) did not reduce femoral BMD and in fact resulted in an increased lumbar BMD [22] (Table 1). In other studies, no reduction in BMD after IVMP therapy was observed: neither in patients with active rheumatoid arthritis (1.0 g for three alternate days, repeated monthly if needed) [20] nor in patients with multiple sclerosis (15 mg/kg for 10 days) [21] (Table 1). However, two reports found a decrease of BMD during IVMP treatment [23, 24] (Table 1). In the Natsui et al. study [23], 19 of 33 patients (a heterogeneous group in respect to underlying diseases and total dosage of GCs) received IVMP followed by oral GCs, while the remaining patients were treated with oral GCs only. A greater decrease in BMD was observed in the lumbar spine than in the femoral neck. In the Haugeberg et al. study [24], 14 of 38 patients with diverse rheumatic disorders received IVMP according to varied regimens, time schedules of pulses and timing of BMD measurements. The decrease of BMD in the femoral neck was conversely greater than the decrease in the lumbar spine. What makes our study unique is that the analysed group of patients was treated for one disease, according to an identical scheme of IVMP pulse therapy, without concomitant oral GCs use, and with BMD evaluation before and after IVMP therapy.

We did not observe any changes in serum Ca, P, and iPTH between baseline and last evaluation. Our data are consistent with previous reports in which no change in Ca, P [21, 27, 28] and parathyroid hormone levels [21, 28, 29] after IVMP therapy was observed.

From a practical point of view, it is worth noting that our study supports the current method of evaluating fracture risk using the FRAX calculator, as it takes into consideration the many factors which may impact risk of fracture (e.g., smoking, alcohol, GCs). GCs should be included if a patient is “currently exposed to oral GCs or has been exposed to oral GCs for more than 3 months at a dose of prednisolone of 5 mg daily or more, or equivalent doses of other GCs” [https://www.sheffield.ac.uk/FRAX/tool.aspx?country=40]. According to our study intravenous GCs given according to a weekly schedule should not be included in this evaluation.

The present study has some limitations. The main weaknesses that should be discussed are the potential effects of pre-treatment hyperthyroidism [30] and vitamin D deficiency on bone metabolism and BMD [31].

Thyrotoxicosis increases bone turnover in favour of bone resorption [32], which results in a decrease of BMD [33]. Numerous studies report a significant increase in BMD within the first years after initiation of antithyroid treatment [33–36]. However, the precise timeline of bone density recovery remains uncertain. The degree of bone loss caused by thyrotoxicosis depends on its severity and duration [37]. All of our patients remained euthyroid at least one month prior to the IVMP therapy as well as during the study. Fourteen patients were either always euthyroid (one patient with euthyroid Graves’) or remained so for at least six months preceding the study (nine patients with Graves’ disease after radical treatment and four patients with Hashimoto disease on levothyroxine supplementation). Twenty-one hyperthyroid patients treated using mainly “block and replace” therapy were euthyroid for at least one month before the study (mean time of euthyroidism was 3 months before treatment with IVMP). All of them became euthyroid within about 4–6 weeks after introducing thyrostatics in the past and stayed euthyroid during further evaluation. We have found no correlations between the duration of euthyroidism before IVMP therapy in those 21 formerly hyperthyroid patients and change in BMD of lumbar spine and femoral neck (Table 6 in the Supplementary Material). There were no differences in duration of euthyroidism before IVMP therapy between subjects with an increase versus those lacking any change in BMD (mean time of euthyroidism was 3 months in both groups) (Table 4). In summary, although it is impossible to exclude the ongoing restoration of BMD after stabilization of thyroid state in former hyperthyroid patients, it is probably not crucial in order to conclude that bone loss recovery after hyperthyroidism remains at least undisturbed.

A high prevalence of vitamin D deficiency is reported in patients with Graves’ disease [38]. Two meta-analysis demonstrated benefits of combined calcium and vitamin D supplementation in the prevention of osteoporosis in patients treated with oral GCs [39, 40]. There is only one previous study assessing the influence of IVMP on BMD [24] in which patients received supplementation with calcium and/or vitamin D. The rate of bone loss among patients in this study had a tendency to be less pronounced in those taking vitamin D than in patients without any osteoporosis prevention. At baseline 51% of our patients were vitamin D deficient (mean 25(OH)D level was 19.3 ng/mL). The precise history of calcium and vitamin D intake before the study was impossible to determine due to the wide availability and use of calcium and vitamin D supplements in patients. All patients received supplementation with 1.0 g of calcium and 800 IU of vitamin D throughout the study. The updated guidelines from the American College of Rheumatology [41] recommend optimizing calcium intake (1.0 g–1.2 g) and vitamin D intake (600–800 IU/day) for all patients receiving treatment with GCs. However, it is crucial to underline that the available guidelines for the prevention of glucocorticoid-induced osteoporosis focus on patients in whom treatment with oral GCs is considered. We observed an increase in 25(OH)D level by the end of the study up to a mean value above 20 ng/mL, which is accepted to be sufficient as far as bone health and calcium homeostasis is considered [31]. We cannot exclude that the lack of decrease in BMD seen in our study may be partially associated with the positive effects of supplementation with vitamin D and calcium. However, we did not find any correlations between changes in lumbar or femoral BMD and baseline 25(OH)D, or changes in the 25(OH)D level (Table 7 in the Supplementary Material). There was no difference between groups with and without increase in BMD as far as the baseline 25(OH)D concentration was considered (Table 4).

The third limitation was reassessment of BMD after a relatively short follow-up period. BMD was measured at the end of the study within three days following the last IVMP pulse. Van Staa et al. demonstrated that the risk of fractures in patients receiving oral GCs increases rapidly within the first months after initiation of treatment and then later declines after stopping therapy [12]. We cannot exclude the possibility of microarchitectural deterioration of bone tissue occurring before bone mass could have been lost. Newer technology in assessing bone microarchitecture, such as trabecular bone score (TBS), could be helpful in estimating bone strength and individual fracture risk in patients on IVMP. Further research with a longer follow-up period and additional measurements of BMD including TBS assessment after 6 or 12 months are needed to determine whether a delayed reduction in BMD develops following pulse therapy or not. Finally, the study was designed with a relatively small number of patients. Our sample size is comparable, however, to previous studies assessing the influence of intravenous GCs on BMD, whose study groups ranged from 13 to 38 patients [20–24] (Table 1).

In summary, we have found that IVMP given in once-weekly pulses during a limited period of twelve weeks (with a cumulative dose of 4.5 g) has no adverse effect on BMD of the lumbar spine and the femoral neck.

## Supplementary information

Supplementary Tables6-7
