# Supplementary material for: Therapy of moderate-to-severe Graves’ orbitopathy with intravenous methylprednisolone pulses is not associated with loss of bone mineral density
Source: Endocrine. 2018 Dec 1;64(2):308–15. doi: 10.1007/s12020-018-1823-x (PMC6531386; doi:10.1007/s12020-018-1823-x)
Supplement: Supplementary file 1 — Supplementary Tables6-7 [file 12020_2018_1823_MOESM1_ESM.docx]

**Table 6** Correlations between the duration of euthyroidism before treatment with IVMP pulses and changes in BMD of the femoral neck and lumbar spine (r)

|  | Change in femoral BMD | Change in lumbar BMD |
| --- | --- | --- |
| Duration of euthyroidism | r = 0.42, *p* value = 0.06 | r = -0.37, *p* value = 0.10 |

Analysis performed in 21 formerly hyperthyroid patients taking antithyroid drugs throughout the study;

BMD–bone mineral density; IVMP–intravenous methylprednisolone; duration of euthyroidism–time between achievement of normal levels of FT4 and FT3 and beginning of therapy with IVMP.

**Table 7** Correlations between 25(OH)D values and changes in BMD of the femoral neck and lumbar spine (r)

|  | Change in femoral BMD | Change in lumbar BMD |
| --- | --- | --- |
| Baseline 25(OH)D | r = -0.19, *p* value = 0.29 | r = 0.07, *p* value = 0.70 |
| Change in 25(OH)D | r = 0.16, *p* value = 0.36 | r = -0.01, *p* value = 0.97 |

Analysis performed in all 35 patients; BMD–bone mineral density; 25(OH)D–25-hydroxyvitamin D; baseline 25(OH)D–level of 25(OH)D before treatment with intravenous methylprednisolone pulses; change in 25(OH)D–change in the level of 25(OH)D during the study
